# Assessing Suitability of a Colorectal Cancer Screening Program in Oman

**DOI:** 10.7759/cureus.27011

**Published:** 2022-07-19

**Authors:** Mohammed Fayyadh Bondre, Murtadha Al Qubtan, Salim O Al Harthy

**Affiliations:** 1 General Surgery, Royal Hospital, Muscat, OMN; 2 Gastroenterology, Royal Hospital, Muscat, OMN

**Keywords:** colorectal cancer, fecal occult blood test, colon cancer prevention, screening programme, oman, bowel cancer screening, colorectal cancer

## Abstract

Colorectal cancer is among the most common cancers globally and carries a high mortality rate. The incidence of colorectal cancer has been increasing in the Middle East, including in Oman. While many countries have implemented a bowel cancer screening program, Oman has yet to establish one. A standard tool for bowel cancer screening is a fecal immunochemical test where a fecal sample is sent to a laboratory to check for blood content in the feces. In Oman, such fecal test kits are available at the primary health care level, but primary care physicians were unaware of the signs and symptoms or screening methods for colorectal cancer. This review article aims to assess the suitability of a colorectal cancer screening program in Oman using guidelines from the Supporting the Use of Research Evidence (SURE) collaboration.

## Introduction and background

**Figure 1:**
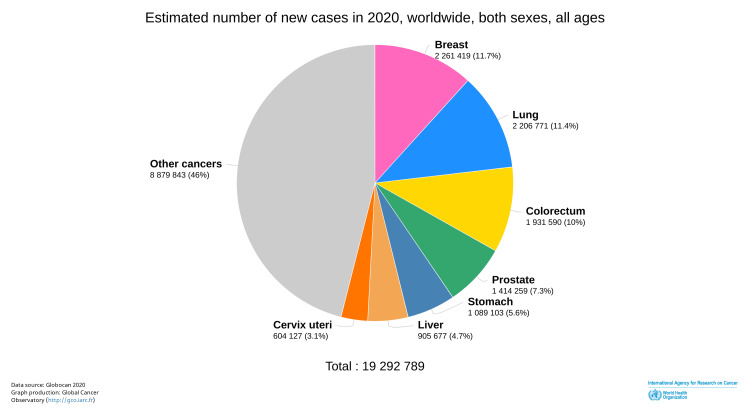
Worldwide incidence of cancers in 2020 ©International Agency for Research on Cancer. Global Cancer Observatory. 2020. Pie chart. Estimated number of new cases in 2020, worldwide, for both sexes, and all ages. https://gco.iarc.fr/today/online-analysis-pie?v=2020&mode=cancer&mode_population=continents&population=900&populations=900&key=total&sex=0&cancer=39&type=0&statistic=5&prevalence=0&population_group=0&ages_group%5B%5D=0&ages_group%5B%5D=17&nb_items=7&group_cancer=1&include_nmsc=1&include_nmsc_other=1&half_pie=0&donut=0. Downloaded June 21, 2022. Reprinted with permission from World Health Organization International Agency for Research on Cancer [2].

**Figure 2:**
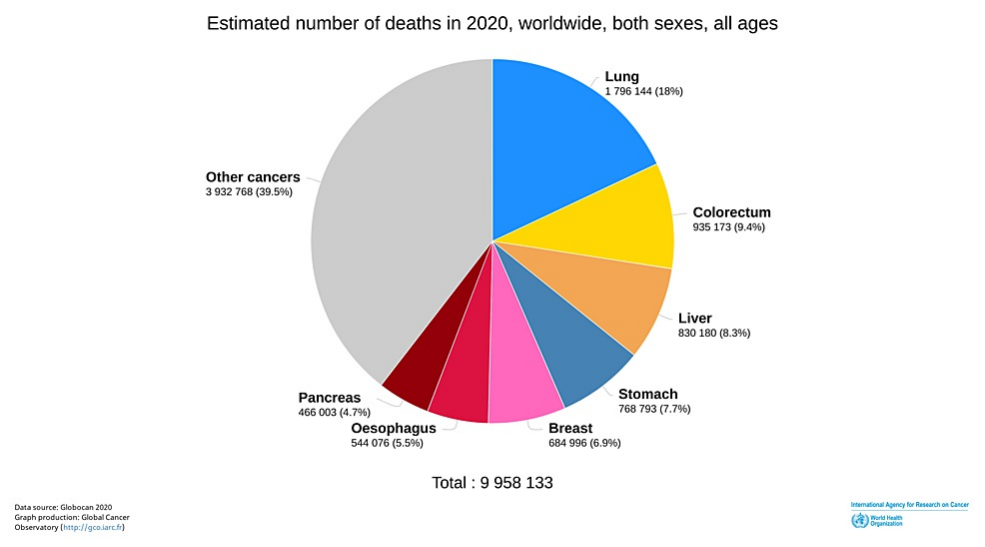
Worldwide mortality due to cancers in 2020 ©International Agency for Research on Cancer. Global Cancer Observatory. 2020. Pie chart. Estimated number of new cases in 2020, worldwide, for both sexes, and all ages. https://gco.iarc.fr/today/online-analysis-pie?v=2020&mode=cancer&mode_population=continents&population=900&populations=900&key=total&sex=0&cancer=39&type=1&statistic=5&prevalence=0&population_group=0&ages_group%5B%5D=0&ages_group%5B%5D=17&nb_items=7&group_cancer=1&include_nmsc=1&include_nmsc_other=1&half_pie=0&donut=0. Downloaded June 21, 2022. Reprinted with permission from World Health Organization International Agency for Research on Cancer [3].

Colorectal cancer (CRC) is cancer of the colon and rectum. In 2016, CRC was reported as the third most common cancer worldwide among both sexes and the fourth leading cause of cancer-related death [1]. Over the next few years, the incidence of CRC increased, and in 2020, CRC remained the third most common cancer among both sexes and became the second leading cause of cancer deaths globally [2,3] (Figures 1, 2).

Oman and its neighboring countries are part of the Gulf Co-operation Council (GCC), a group of high-income countries (HICs) with similar religious and cultural habits. These countries witnessed rapid industrialization and development over the past 40 years. During that time, residents of these countries also changed their lifestyles and dietary habits. Decreasing levels of physical activity, increased obesity, increased uptake of smoking, and unhealthy diets are some of the factors related to developing CRC among Arabs and Omanis [4,5].

Although the countries in the GCC are considered low-risk for CRC, a review of the incidence of CRC in the Middle East by Alhurry et al. reported that the incidence of CRC was increasing, and the highest incidence rate of CRC among the GCC nations was in Kuwait, followed by Saudi Arabia [6].

Like its GCC neighbors, Oman has witnessed a steady increase in CRC incidence from 1996 to 2015 [7]. As per the 2018 World Health Organization (WHO) country cancer profile, CRC was the second most common cancer in Oman and the highest cause of cancer deaths [8]. Recent data show that CRC is the most commonly diagnosed cancer in men and the third most common cancer in women in Oman [9].

Fadhil et al. assessed the rising CRC incidences in the member countries of the GCC and advised the introduction of a CRC screening program in a phased manner [10]. HICs like the United Kingdom have a national CRC screening program where a patient is given a kit called fecal immunochemical test (FIT) [11]. The patient uses this to collect a fecal sample and send it to a laboratory to check for fecal blood. If the test detects blood, the patient is then contacted for further screening by colonoscopy.

Qatar has implemented similar screening programs for its population [12]. As of this writing, a screening program for CRC does not exist in Oman despite the recent growing incidence of CRC in the country. This undoubtedly increases the financial burden on the government of Oman. There are specific criteria for screening programs, such as the Bradford Hill criteria. This review article aims to assess the suitability of a CRC screening program in Oman using guidelines from the Supporting the Use of Research Evidence (SURE) collaboration [13].

## Review

Criteria 1: Is the health problem important?

**Figure 3:**
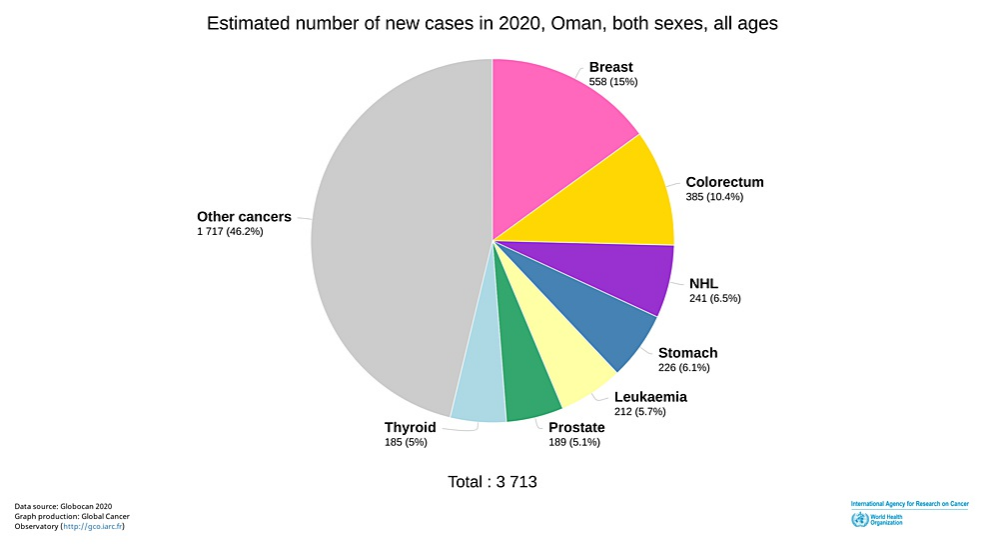
Incidence of cancers in Oman in 2020 ©International Agency for Research on Cancer. Global Cancer Observatory. 2020. Pie chart. Estimated number of deaths in 2020, Oman, for both sexes, and all ages. https://gco.iarc.fr/today/online-analysis-pie?v=2020&mode=cancer&mode_population=continents&population=900&populations=900_512&key=total&sex=0&cancer=39&type=0&statistic=5&prevalence=0&population_group=0&ages_group%5B%5D=0&ages_group%5B%5D=17&nb_items=7&group_cancer=1&include_nmsc=1&include_nmsc_other=1&half_pie=0&donut=0#collapse-by_country. Downloaded June 21, 2022. Reprinted with permission from World Health Organization International Agency for Research on Cancer [9].

As reported by the Global Cancer Observatory (GLOBOCAN), the incidence of CRC in Oman has risen to the most diagnosed cancer among men and the second most common cancer among both sexes in Oman (Figure 3) [9]. The incidence and prevalence of CRC have increased over the past 10-15 years (as of this writing) [4,5].

**Figure 4:**
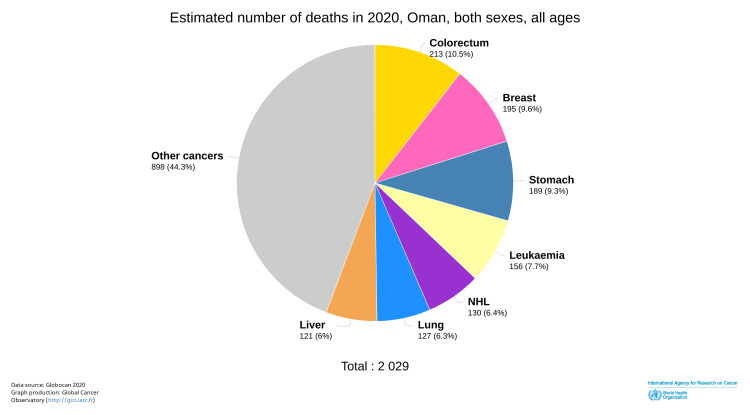
Mortality due to cancers in Oman in 2020 ©International Agency for Research on Cancer. Global Cancer Observatory. 2020. Pie chart. Estimated number of deaths in 2020, Oman, for both sexes, and all ages. https://gco.iarc.fr/today/online-analysis-pie?v=2020&mode=cancer&mode_population=continents&population=900&populations=900_512&key=total&sex=0&cancer=39&type=1&statistic=5&prevalence=0&population_group=0&ages_group%5B%5D=0&ages_group%5B%5D=17&nb_items=7&group_cancer=1&include_nmsc=1&include_nmsc_other=1&half_pie=0&donut=0#collapse-by_country. Downloaded June 21, 2022. Reprinted with permission from World Health Organization International Agency for Research on Cancer [15].

Studies have also reported that despite CRC affecting mainly older age groups, CRC in Oman has been diagnosed in younger patients (i.e., those younger than 40 years), and patients often present with advanced stages of the disease [14]. Mortality due to CRC is the highest among all cancers in Oman (Figure 4) [15].

Presently, Omani nationals have free healthcare paid for entirely by the government. The burden of CRC is expected to increase significantly by 2040, and Oman is no exception [16]. Actual expenses incurred by the Omani government due to CRC are not disclosed, but a rough estimate regarding the burden of CRC could be calculated by comparing the expenditure on CRC management by another HIC country - the United States of America (USA). Average Medicare expenditure on a patient with CRC varies between $60,000 and $73,000 per year, depending on the stage of the CRC [17]. CRC management costs an estimated $14.1 billion annually in the USA alone [18]. As with other cancers like breast or cervical cancers, the WHO recommends implementing CRC screening programs, and early detection effectively reduces the cancer burden on a country [19]. Previous studies advocate for Oman to adopt a CRC screening program [5,20,21].

Criteria 2: Are viable options available to address the problem?

There are multiple screening tools and tests for CRC. Three fecal/stool tests are available: the guaiac fecal occult blood test (gFOBT), the FIT, and the multitargeted DNA stool test (FIT-DNA). The gFOBT uses a feces sample to detect heme, a component of hemoglobin. gFOBT can detect heme in certain foods like red meats, so patients should avoid heme-rich foods before performing this test. The FIT kit uses an antibody to detect the hemoglobin protein in the fecal sample. The FIT-DNA test kit detects DNA biomarkers and hemoglobin in the fecal sample. When stool passes through the colon, some of the colon's mucosa cells are shed along with it. If this mucosa contains cancerous cells, the DNA of the tumor will be detected.

The specificity for these tests is high, ranging between 96.8% and 98.6%, but the sensitivity for advanced CRC is high in the FIT kits [22]. The sensitivity of the FIT-DNA kits is the highest, followed by the FIT and gFOBT kits. However, FIT-DNA tests yield more false positives in asymptomatic patients, which could lead to additional unnecessary colonoscopies [23].

Other screening tests for CRC are colonoscopy and computed tomography (CT) colonography. Colonoscopy is considered the gold-standard diagnostic modality for screening for CRC because this allows for visual confirmation of bowel tumors and access to biopsy the bowel mass/lesion. The main complication of this procedure is the possibility of colon perforation during the procedure. Another alternative screening test for CRC is the use of CT colonography. In this procedure, a small tube is inserted into the rectum to inflate the colon with air. The CT machine then scans the patient. The drawback of this procedure is that if suspicious polyps are identified on the scans, the patient will need a colonoscopy procedure to have these removed and sent to the histopathology laboratory for confirmation of the benign or malignant nature of the polyp.

In Oman, primary healthcare centers all over the country are equipped with gFOBT kits [5]. Colonoscopy is performed in most tertiary care hospitals in Oman. A CRC screening campaign with FIT kits over three days was held in 2019 by the Colorectal Surgery Unit of the Royal Hospital, Muscat [24]. This campaign had garnered a good response.

The screening modalities listed above are all readily available in Oman. Like other HIC countries and Qatar, Oman could use gFOBT to screen patients aged 50-75 for CRC. Patients with positive results could be referred to the tertiary center for colonoscopy.

Criteria 3: Is there an opportunity for change?

Awareness of CRC screening among the Omani population has been documented to be low [21]. This increased slightly after a public campaign for CRC screening by test kits at a tertiary hospital was held in Muscat in December 2019. Like many other countries, Oman is attempting to get back to regular day-to-day life schedules as before the coronavirus disease 2019 (COVID-19) pandemic.

**Figure 5:**
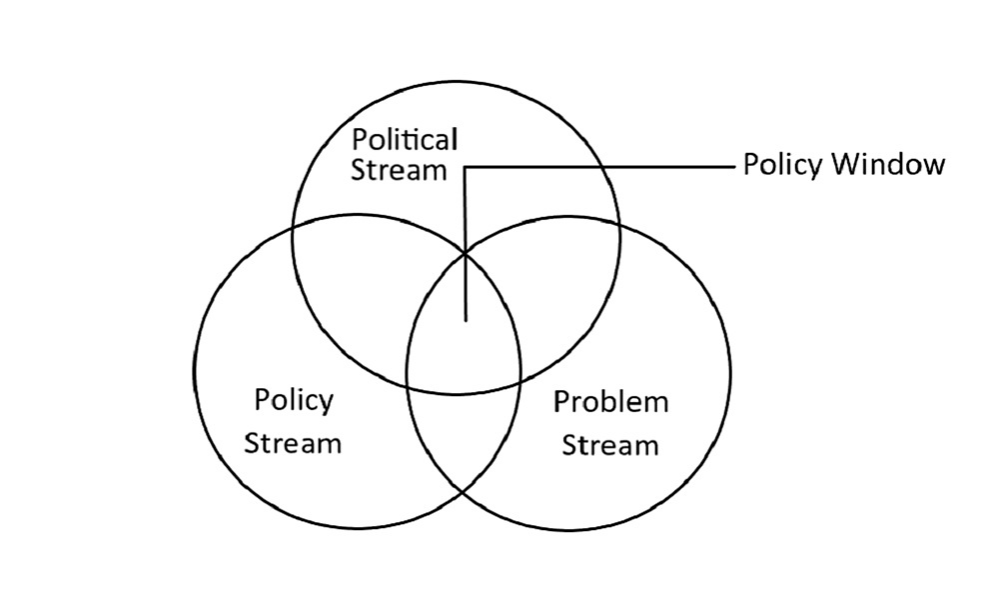
Kingdon's multipolicy stream model Adapted from Kingdon: Agendas, Alternatives, and Public Policies. Updated Second Edition [25].

Given the rising incidence of CRC in the region and Oman, the high mortality in cancer deaths in the country, and a possible increase in awareness of CRC, a policy window might exist currently to persuade the Oman Ministry of Health to adopt national screening for CRC. Kingdon's multipolicy streams model could be applied in this setting, as shown in Figure 5 [25].

The problem stream is the high incidence of CRC and CRC mortality in Oman, the low awareness of the population regarding CRC, and the availability of trained personnel in the healthcare sector. The political stream consists of administrators at the Ministry of Health, and the policy stream consists of multidisciplinary teams of doctors and nongovernment organizations (NGOs) who could emphasize the need for a CRC screening program in the country.

Criteria 4: Is there important uncertainty about the problem and potential solutions?

Qatar's CRC screening program commenced in 2016 as per the policies laid by the National Cancer Program [12]. Patients aged 50-74 were issued an FIT kit from primary healthcare centers. If a patient obtained a positive result, they would undergo a colonoscopy within 30 days. A patient could also obtain a positive result on an FIT if they have other conditions like bleeding colon diverticula, polyps, hemorrhoids, or irritable bowel syndrome.

A cross-sectional study by Al-Dahshan et al. reported low awareness of CRC risk factors and symptoms among the target population in Qatar despite a screening program that commenced in 2016 [26]. Similarly, public awareness about CRC in Saudi Arabia is also low [27,28]. This trend was also noted in Oman [21]. Saudi Arabia has published guidelines for screening for CRC but still does not have a national screening program [29].

One issue the Oman healthcare system faces is the long waiting list for colonoscopy and surgical operation appointments. Patients from different regions of Oman insist on being treated in the main tertiary hospital of the country located in the capital, Muscat - the Royal Hospital. The colorectal surgery unit is present in the Royal Hospital only. Therefore, the Royal Hospital became a focal point for CRC referrals and management.

Another issue the healthcare system faces is a shortage of operating theaters. The recent pandemic worsened the shortage when elective surgery cases were canceled or postponed, and only select patients with advanced CRC disease received surgical operations. With the intensive care units (ICUs) around the country saturated with COVID-19 patients, booking beds in the ICU for advanced CRC patients who required surgery represented a considerable challenge.

Several issues must be addressed. The level of CRC awareness among the public needs to be increased. Media campaigns are required to educate the public on screening programs, especially the older age group of patients aged 50-75. Physicians at the primary healthcare centers need to be trained to be more vigilant when treating patients in this age group and offer testing kits to suspected patients. Besides the availability of test kits, follow-up of patients with positive results and fast-tracking their colonoscopy appointments are required. These measures will need considerable labor force and financial support.

A multidisciplinary team consisting of stakeholders - medical personnel (e.g., surgeons, gastroenterologists, pathologists, primary care physicians, nurses), patients, and NGOs (e.g., the WHO) - would need to cooperate and meet with officials from the Ministry of Health to emphasize the issue of CRC and its shared burden. Only through these persistent discussions could a policy window be created, and a national screening program for CRC in Oman could commence.

Criteria 5: Is relevant research evidence available?

Published studies have confirmed that screening for CRC reduces a country's cancer burden [19,30]. Even though the awareness of CRC was low internationally and in Oman and its neighboring countries, studies emphasize that more needs to be done to reduce the incidence of CRC by adopting a screening program and increasing awareness to improve the population's health.

The population must be educated that the primary prevention of CRC is by adopting healthier diets, including additional vegetables and fibers, and increasing physical activity. Secondary prevention is possible by adopting a screening program to look for and eliminate precancerous lesions using fecal tests followed by a colonoscopy if the result is positive [15]. Screening programs for CRC have been shown to reduce CRC mortality in various countries and thereby reduce the burden of cancer on a country [30].

Criteria 6: Is there interest in informed deliberation about the problem and potential solutions?

Although patients' awareness of CRC in Oman was low, they were willing to undergo a screening test if advised by their doctor [20]. In the same study, the authors identified emotional and cultural barriers that older Omani men and women faced regarding CRC screening and advised further efforts to tackle these issues. As suggested earlier, a multidisciplinary team of stakeholders should meet with Oman's Ministry of Health officials to discuss campaigns to raise the population's awareness regarding CRC and the adoption of a screening program. These measures will reduce the CRC burden on Oman, enable people to live longer and healthier lives, and reduce the incidence of CRC in Oman.

## Conclusions

Oman has witnessed an increase in the incidence of CRC and CRC-related death. A CRC screening program has not yet been implemented in Oman, but some neighboring countries have adopted such a program. Using the SURE guidelines, we assessed the suitability of a CRC screening program and identified a few challenges that could be encountered when implementing such a program in Oman. Some cultural and emotional barriers to accepting screening tests and diagnosing CRC exist. Physicians' awareness of CRC and its screening at the primary healthcare level was low. To reduce the cancer burden on Oman, a campaign to educate the population and doctors at the primary healthcare level should be launched to increase awareness of CRC. Following this, national screening guidelines must be published, and a CRC screening program must be adopted.
